# The Role of Radiotherapy in the Management of Melanoma Brain Metastases: An Overview

**DOI:** 10.1007/s11864-024-01289-y

**Published:** 2025-01-03

**Authors:** Marko Lens, Jacob Schachter

**Affiliations:** 1 University of Leeds, Beckett Street, Leeds, LS9 7TF UK; 2 Ella Lemelbaum Institute for Immuno Oncology, Chaim Sheba Medical Center, 6997801 Tel Aviv, Israel

**Keywords:** Melanoma, Brain metastases, Radiation, Stereotactic radiosurgery

## Abstract

Clinical management of melanoma brain metastases is complex and requires multidisciplinary approach. With close collaboration between neurosurgeons, radiation oncologists and medical oncologists, melanoma patients with brain are offered different treatment modalities: surgery, radiation therapy, systemic therapy or combined treatments. Radiation therapy (whole brain radiotherapy- WBRT and stereotactic radiosurgery- SRS) is an integral part of treating melanoma brain metastases. Use of immunotherapy (checkpoint inhibitors) and targeted therapy (BRAF/MEK inhibitors) significantly changed the outcome in patients with melanoma metastases. Currently, ipilimumab and nivolumab (COMBO) is the preferred first-line systemic therapy for all patients with asymptomatic brain metastases, regardless of BRAF status (BRAF wild-type and BRAF-mutated). Although at the moment there is no consensus on the concomitant use of SRS and COMBO, results from clinical trials suggest that this combined treatment modality should be considered the standard of care for melanoma patients with brain metastases. However, further clinical research is required to define optimal treatment modalities for routine management of melanoma brain lesions.

## Introduction

Cutaneous melanoma represents the most aggressive type of skin cancer due to its high potential for metastatic spread. While the incidence of primary melanoma continues to progressively increase in Europe, latest data suggest that the rates of melanoma incidence have levelled off in North America and Oceania [1].

Melanoma is the third most common primary malignancy that leads to brain metastases (5–20%) and together with lung cancer (40–50%) and breast cancer (15–30%) accounts for up to three-quarters of metastatic brain lesions [2]. Thus, among solid tumours melanoma has the highest predilection for metastatic dissemination to the brain. Although clinical data are showing that up to 50% of patients with stage IV melanoma are developing brain metastases at some point in the course of their disease, results from autopsy are revealing involvement of the brain in over 75% of patients with metastatic melanoma [3]. Brain metastases are associated with significant morbidity and mortality. Currently several treatment options are available for melanoma patients with brain metastases which include surgery, targeted therapy, immunotherapy and radiotherapy. Until recently, brain metastases have been the leading cause of death from melanoma with the median survival of patients diagnosed with brain metastases of only about 4 months [4]. However, the use of systemic therapy including immunotherapy (immune checkpoint inhibitors) and targeted therapy (combination of BRAF/MEK inhibitors) improved the tumour response and survival in patients with melanoma brain metastases. Clinical evidence suggests that local tumour response and overall survival are significantly lower in patients with symptomatic melanoma brain metastases (response rates of 15%–20% with median overall survival < 9 months) comparing to those with asymptomatic disease (response rates of 50%–60% with median overall survival > 5 years) [5]. While the surgical resection of brain lesion remains the gold standard for the local tumour control in patients with a solitary and symptomatic brain metastasis, continuous advances in radiation therapy opened new treatment options offering improved clinical outcomes for patients with melanoma brain metastases. Treatment of patients with brain metastases is complex and requires multi-disciplinary approach.

The objective of this review is to provide an overview of currently available radiation therapy modalities and discuss their role and future perspectives in the management of melanoma brain metastases.

Two main radiation therapy (RT) modalities used for the treatment of melanoma brain metastases are whole brain radiation therapy (WBRT) and stereotactic radiosurgery (SRS).

## Whole Brain Radiotherapy (WBRT)

The whole-brain radiation therapy (WBRT) administered alone has been traditionally considered the standard treatment in all patients with multiple melanoma brain metastases. WBRT has been also prescribed after surgical resection or radiosurgery of brain metastases with an aim to reduce the risk of local relapse and to improve distant brain control or as palliative treatment to alleviate symptoms from brain metastases. Although different WBRT protocols have been described, two most commonly used standard dose-fractionation regimens for melanoma brain metastases were 30 Gy in 10 fractions or 35 Gy in 14 fractions [6]. The major adverse effect of conventional WBRT is neurocognitive toxicity caused by radiation-induced injury to the hippocampus. It has been reported that a neurocognitive decline occurs in up to 90% of patients surviving more than 6 months after WBRT [7]. The cognitive decline primarily reflecting verbal and short-term memory recall usually develops within 4 months after WBRT, seriously affecting the quality of life of patients receiving this form of radiation therapy. The risk of occurrence of neurocognitive toxicity depends of the total radiation dose, the dose-per-fraction and radiation dose received by the hippocampus [8]. In order to preserve a memory-specific neural stem cell compartment in the hippocampus and thus mitigate the neurocognitive deficits associated with standard WBRT, diverse radiation strategies have been explored. In 2010, Gondi et al. proposed the brain irradiation with hippocampal sparing to reduce development, severity and frequency of neurocognitive decline in melanoma patients undergoing WBRT for brain metastases [9]. They suggested helical tomotherapy and linear accelerator (LINAC)-based modern intensity-modulated radiotherapy (IMRT) technology with avoidance of hippocampus occupying 2.1% of the whole-brain planned target volume. While helical tomotherapy spared the hippocampus with a median dose of 5.5 Gy and maximum dose of 12.8 Gy, LINAC-based IMRT spared the hippocampus with a median dose of 7.8 Gy and maximum dose of 15.3 Gy. Thus, helical tomotherapy and LINAC-based IMRT for a prescription dose of 30Gy in 10 fractions reduced the mean dose per fraction to the hippocampus (normalized to 2-Gy fractions) by 87% (to 0.49 Gy_2_) and 81% (to 0.73 Gy_2_), respectively. Based on achieved results a phase II multi-institutional clinical trial (RTOG 0933) was developed to evaluate the neurocognitive benefit of WBRT with conformal hippocampal avoidance (HA-WBRT) [10]. To compare results of patients treated with HA-WBRT this trial used a prespecified historical control composed of patients with brain metastases treated with standard WBRT (30 Gy in 10 fractions without hippocampal avoidance) included in the control arm of the PCI-P-120–9801 phase III trial [11]. Results from RTOG 0933 trial published in 2014 showed that hippocampal sparing during WBRT of brain metastases is associated with significant preservation of memory functions and no decline in quality of life [12]. Cognitive assessment performed using the Hopkins Verbal Learning Test–Revised Delayed Recall (HVLT-R DR) test at baseline (before radiation) and 4 months after radiation showed that the achieved scores were significantly higher in patients treated with HA-WBRT comparing to those from the historical control (30% vs 7%, *p* < 0.001), indicating a clear memory benefit of hippocampal avoidance. Later on, a secondary analysis of RTOG 0933 data supported the importance of WBRT-induced intracranial control on neurocognition [13]. This analysis showed that the volumetric area of white matter injury (WMI) before the radiation treatment detected using MRI FLAIR (Fluid-attenuated inversion recovery detected by Magnetic resonance imaging) as a surrogate of injury, predicts the post-radiation injury and correlates with memory decline after HA-WBRT. Based on promising results from RTOG 0933, a phase III NRG Oncology CC001 trail has been conducted with an aim to assess the time to cognitive function failure in patients treated with WBRT plus.

Memantine (NMDA receptor antagonists used as neuroprotector for Alzhemier’s disease) with or without hippocampal avoidance. At the median follow up exceeding 12 months although published results failed to show significant difference in intracranial progression-free survival and overall survival in patients treated with HA-WBRT plus memantine versus those treated with WBRT plus memantine, this trial demonstrated that the addition of HA (hippocampal avoidance) to WBRT significantly reduced cognitive failure risk (unadjusted HR = 0.76, 95%CI: 0.60–0.98, *p* = 0.029) confirming again the importance of hippocampal sparing in preserving neurocognitive function [14].

In the light of the fact that the hippocampal avoidance is recommended to preserve the radiosensitive, memory-specific neural stem compartment with great neuronal regeneration and plasticity rather than any anatomic component of hippocampus, HA-WBRT has become a routine radiation technique of choice in the management of brain metastases in patients with good performance status who are considered for WBRT [15, 16]. In recent years, technological advances in radiotherapy led to the development of modern techniques for hippocampal sparing: photon RT techniques (Intensity-modulated arc therapy- IMAT and Volumetric arc intensity-modulated RT- VMAT), Spiral Tomo and Proton RT techniques [17]. However, the efficacy and advantages of these techniques comparing to conventional IMRT still have to be established in clinical trials.

The efficacy of WBRT in patients with brain metastases has been questioned for many years, particularly due to the fact that melanoma has been traditionally considered a radioresistant tumour.

Results from meta-analysis including 11,898 patients from 54 clinical trials evaluating the effectiveness and adverse effects of WBRT in the management of multiple brain metastases found no benefit of WBRT in terms of overall survival [17].

In 2019 Hong et al. published the results from the largest phase III multicenter, randomised trial including 215 patients who were after local treatment of one to three melanoma brain metastases by surgery and/or SRS randomly assigned to WBRT or observation [18]. At the median follow-up of 48.1 months this study failed to show that adjuvant WBRT in comparison to observation provides any significant clinical benefit in terms of the distant intracranial control rate (48% vs 42.1%, *p* = 0.39), median overall survival (16.5 months vs 13 months, *p* = 0.86) or median time of preservation of performance status (3.8 months vs 4.4 months, *p* = 0.32). Although WBRT has been the mainstream treatment for many patients with multiple brain metastasis, clinical results showed that use of conventional WBRT in patients with multiple brain metastases provides a poor local tumour control and a median survival of only 3 to 4 months [17, 19].

Insufficient clinical efficacy and high neurocognitive toxicity of WBRT prompted the development of new radiotherapy strategies. To improve the local tumour control a lesion-targeted radiation boost has been added to WBRT and thus the WBR plus simultaneous integrated boost (SIB-WBRT) and WBRT plus sequential integrated boost (SEB-WBRT) have been introduced.

A randomized two-armed study (DRKS00005127) that recruited only 7 patients with melanoma brain metastases at the median follow-up of 5 months showed promising results suggesting that HA-WBRT + SIB may improve local tumour control [19]. Sadly, this trial ended early due to poor recruitment.

A single-arm phase II trial including 50 patients evaluating the efficacy of HA-WBRT with simultaneous integrated boost- SIB (HSIB-WBRT) at the mean follow-up of 10.5 months showed that median progression-free survival of 2.9 months and median overall survival of 9 months were comparable to those observed in historical controls (20Patients included in this trial had 1-year cumulative incidences of local failure and intracranial failure of 8.8% (95% CI: 2.7‒19.6%) and 21.3% (95% CI: 10.7‒34.2%), respectively [20]. However, about 90% of patients included in the study had non-melanoma brain metastases.

Results from a cohort study including 62 patients treated with HA-WBRT + SIB and 62 patients treated with WBRT showed that in comparison to WBRT patients undergoing HA-WBRT + SIB had significantly improved intracranial progression-free survival (3.5 months vs 6.4 months, *p* = 0.03) and overall survival (9.9 months vs 6.2 months, *p* = 0.001) [21]. At 1 year of follow up, patients treated with HA-WBRT + SIB achieved significantly better local tumour control comparing to those who had WBRT (98% vs 82%*, p* = 0.007), while due to higher biologically effective doses WBRT led to significantly better distant intracranial tumour control (82% vs 69%, *p* = *0*.016). This study included only 17 patients (12 in HA-WBRT + SIB group and 7 in WBRT group) with brain metastases due to primary melanoma.

In a retrospective analysis of 83 patients who received WBRT alone and 82 patients who received SIB-WBRT, Zhang et al. showed that SIB-WBRT improved local tumour control and intracranial progression-free survival in comparison to WBRT alone [22]. However, over 95% of analysed patients had brain metastases due to non-melanoma primary tumours (NSCLC, SCLC and breast cancer).

In a retrospective analysis including 468 patients with brain metastases Dobi et al. evaluated efficacy and toxicity of conventional WBRT versus WBRT + consecutive, delayed or simultaneous integrated boost (SIB) [23]. In 46 patients with melanoma brain metastases overall survival was significantly better among who received escalated total dose comparing to those treated with WBRT only (6.5 months vs 3.2 months, *p* = 0.03). This study found that the simultaneous delivery of WBRT with reduced fraction dose and boost is associated with reduced probability for cognitive decline in all patients including those with poor performance status.

Currently there is no sufficient evidence to justify the use of HA-WBRT with or without SIB over WBRT alone.

A phase II, randomised HIPPORAD trial (DRKS00004598) with 136 included patients evaluated the impact of hippocampal sparing on the neurocognitive function comparing HA-WBRT + SIB versus WBRT + SIB in patients with 4 to 10 brain metastases ≥ 5mm diameter [24]. Awaited results from this trial will answer whether the combination of hippocampus avoidance and a lower whole-brain dose may prevent neurocognitive decline while achieving acceptable clinical results.

## Stereotactic Radiosurgery (SRS)

Stereotactic radiosurgery (SRS) involves technology that delivers multiple convergent beams of high radiation (gamma rays, x-rays or protons) to a targeted, radiographically defined treatment region.

Since Lars Leksell introduced the concept of SRS in 1951, many different technologies have been developed to deliver SRS treatment. The most used radiosurgery systems in the treatment of melanoma brain metastases were Gamma Knife (frame technology) and CybeKnife (frameless technology).

Radiosurgery of brain metastatic melanoma initiated in early 90s and since these early starts the role of SRS in the management of melanoma significantly evolved. In recent years we experienced a clear trend of replacing surgery with SRS and favouring SRS over conventional WBRT has been observed. Many clinical studies were performed to assess safety and efficacy of SRS in terms of local control and survival in melanoma patients with brain metastases.

At the median follow-up of 6.8 months of 122 patients with brain metastases treated with gamma knife over the period of 5 years, in 2002 Yu et al. reported the overall median survival of 7 months [25]. In this study significantly improved survival has been observed in patients with the total brain tumour volume of less than 3 cm^3^ (*p* = 0.003) and no presence of active systemic disease (*p* = 0.0065). In a review of 244 patients who underwent gamma knife radiosurgery for 754 brain metastases, Mathieu et al. in 2007 showed that gamma knife radiosurgery provided local control in 86.2% of tumours while offering the median survival of 5.3 months after the gamma knife treatment (range 0.2 to 114.3 months) [26]. A retrospective review published by Liew et al. in 2010 showed that the local tumour control was achieved in 73% of 333 melanoma patients who had gamma knife radiosurgery for 1570 brain metastases while the prolonged median survival was observed in patients who had ≤ 8 brain metastases, RPA (Recursive Partitioning Analysis) Class I and no previous WBRT [27]. Neal et al. in 2014 in the study assessing potential predictive factors of the efficacy of gamma knife radiosurgery performed in 129 patients with melanoma brain metastases reported median survival of 6.7 months and at 12 months after radiosurgery treatment achieved local tumour control and freedom from distant failure in 81% and 29% of evaluated patients, respectively [28].

In 2018 Matsunaga et al. published the results from a retrospective cohort study conducted by the Japanese Leksell Gamma Knife Society (JLGK1501) including 177 melanoma patients with 1,500 brain metastases treated with gamma knife radiosurgery [29]. At 12 months after radiosurgery, they observed the median survival of 7.3 months and local tumour control in 86.2% of patients. This study findings indicated that larger tumour volume and intratumoral haemorrhage had a significant negative impact on the local tumour control.

In an extensive systematic review by pooling the data from different studies including patients with melanoma brain metastases, Thompson et al. reported the median survival of 7.5 months (range, 6.7 months to 9.0 months) in 1188 patients from 8 studies treated with SRS and 3.5 months (range 2.4 months to 4.0 months) in 2185 patients from 26 studies treated with WBRT alone [30]. The 1-year survival was significantly better in in 330 patients from 6 studies treated with SRS alone (35.5%, range 20.8% to 47.8%) comparing to 189 patients from 7 studies in which metastases were treated with WBRT alone (9%, range 0 to 22.5%). Data from 4 studies including 260 patients with brain metastases treated with SRS alone showed the highest 1-year local tumour control rate (76%, range 62.8% to 88.5%).

Based on available clinical evidence showing that radiosurgery improves local tumour control and survival, SRS has emerged as a major alternative to surgery and a replacement of WBRT in all patients with ≤ 4 asymptomatic brain metastases [31].

Management of melanoma patients with high number of brain metastases using SRS is still subject to discussion. A retrospective cohort study of 75 patients with ≥ 20 brain metastases treated with a single-session SRS reported the median overall survival of 4.1 months after the treatment in 14 patients with melanoma [32]. Bowden et al. reported that gamma knife SRS represents a viable treatment option in the management of extensive brain metastases since it delivers a relatively low dose of radiation to the brain comparing to the traditional WBRT [33]. While SRS provides improved local tumour control without the neurocognitive side effects associated with WBRT, Riina et al. are warning that treatment planning of gamma knife SRS in patients with extensive brain metastases (≥ 10 lesions) may need proper hippocampal dosimetry and evaluation of the need for hippocampal-sparing [34].

Since SRS became a standard option for the treatment of melanoma brain metastases while immunotherapy (immune checkpoint inhibitors) and targeted therapy (BRAF/MEK inhibitors) significantly improved outcomes and prognosis of patients with advanced melanoma, over past years concurrent administration of SRS with immunotherapy or targeted therapy has been intensively evaluated in clinical management of melanoma brain metastases [35, 36]. Data from numerous retrospective analyses showed that the application of immunotherapy or targeted therapy concurrently with SRS in patients with melanoma brain metastases is safe and provides a clear advantage in terms of the time to disease progression and survival [37, 38]. Many systematic reviews reported that combination of SRS with immunotherapy or targeted therapy improves local tumour control and provides survival benefit in melanoma patients with brain metastases [39–47]. Based on the currently available evidence local brain therapy with SRS plus systemic immunotherapy (combination of anti-CTLA-4 and anti-PD-1) is considered the most recommended treatment option for patients with asymptomatic melanoma brain metastases [48].

Management of patients with melanoma brain metastases still remains complex and challenging. Optimal treatment planning requires multidisciplinary approach with involvement of surgical oncologists, radiation oncologists and medical oncologists. Currently, different radiotherapy modalities are used in daily management of patients with melanoma brain metastases.

In recent years there was a clear increase of the use of stereotactic radiosurgery (SRS) since it has emerged as a major alternative to invasive surgery and conventional whole brain radiotherapy (WBRT) associated with serious neurocognitive toxicity. Thus, nowadays according to different guidelines SRS became a mainstay in the treatment of melanoma patients with brain metastases.

Evaluation of guidelines provides important recommendations in respect of: 1) use of SRS alone; 2) use of concurrent SRS and immunotherapy/targeted therapy and 3) use of WBRT alone.

### Recommendations Regarding Use of SRS Alone

The European consensus-based interdisciplinary guideline, established as a result of collaboration of the European Organization for Research and Treatment of Cancer (EORTC), the European Association of Dermato-Oncology and the European Dermatology Forum (EORTC guideline) recommends a single-dose SRS to all melanoma patients with brain metastases as the first-line treatment and reserves surgery as a treatment option only when SRS is not possible [49]. Although EORTC guideline recommends SRS to patients with up to 5 brain lesions and diameter of ≤ 3 mm, according to the latest update of this guideline SRS can be considered also in selected patients with larger number of brain lesions [50].

EANO-ESMO guideline developed by the European Association of Neuro-Oncology and the European Society for Medical Oncology (ESMO) suggest SRS as preferred treatment local treatment for asymptomatic patients with a limited number (1 to 4) of brain metastases with a maximum diameter of 4 cm or higher number (5 to 10) of brain metastases with the maximum diameter of 3 cm and a cumulative tumour volume ≤ 15 ml [51, 52].

While evidence-based guidelines provided by the Australian Cancer Council (CCA) recommend surgical resection of brain metastases is recommended for metastases in non-eloquent areas ≥ 1cm or symptomatic metastases, these guidelines consider SRS for single or small number of asymptomatic brain metastases (≤ 3 in diameter) to maximise local tumour control [53].

The US National Comprehensive Cancer Network (NCCN) guidelines considers SRS the primary treatment for limited and multiple asymptomatic melanoma brain metastases [54]. This is in line with ASCO-SNO-ASTRO (American Society of Clinical Oncology–Society for Neuro-Oncology–American Society for Radiation Oncology) guideline which recommends SRS for all patients with up to 10 brain metastases [55]. It appears that in the decision regarding upfront therapy of brain metastases with SRS the lesion volume is more critical than absolute number of brain metastases.

All guidelines recommend SRS delivered to the resection cavity after complete or incomplete resection of melanoma brain metastases.

### Recommendations Regarding Use of Concurrent SRS and Immunotherapy or Targeted Therapy

Use of checkpoint inhibitor immunotherapy inhibitors led to significant improvement of overall survival of melanoma patients and dramatically changed the standards of systemic therapy in the management of patients with advanced melanoma, particularly those with brain metastases. EORTC, EANO-ESMO, CCA and NCCN guidelines recommend combination immunotherapy with ipilimumab and nivolumab (COMBO) as the preferred first-line systemic therapy for all patients with asymptomatic brain metastases, regardless of BRAF status (BRAF wild-type and BRAF-mutated).

The standard COMBO protocol includes the use of the anti-cytotoxic T-lymphocyte-associated protein 4 (CTLA-4) monoclonal antibody ipilimumab (3 mg/kg) and the anti-programmed cell death protein 1 receptor (PD-1) checkpoint inhibitor nivolumab (1 mg/kg) every 3 weeks, 4 doses. Several retrospective and prospective studies evaluated the efficacy and safety of COMBO immunotherapy combined with SRS in melanoma patients with brain metastases.

A retrospective study including 376 melanoma patients with brain metastases treated with COMBO outside clinical trials assessed clinical impact of concomitant radiotherapy (radiotherapy performed within two weeks of starting or ending immunotherapy) and sequential radiotherapy (in other cases) [56]. Results showed significantly better median overall survival in patients who received COMBO and SRS (concomitant or sequential) than in those receiving WBR (30.5 months versus 18.2 months respectively, *p* < 0.0001). The results from this study revealed that patients treated with COMBO + SRS versus those receiving COMBO alone had significantly better median overall survival: in asymptomatic patients (47.0 months versus 20.5 month respectively, *p* = 0.016) while in symptomatic patients (16.4 months versus 9.6 months respectively, *p* = 0.013).

A meta-analysis including 1356 patients with brain metastases from 16 retrospective studies evaluated efficacy of immune checkpoint inhibition (ICI) therapy with SRS as concurrent therapy (ICI and SRS being administered within 4 weeks one after the other) versus nonconcurrent therapy (either ICI more than 4 weeks before or more than 4 weeks after SRS) [57]. The results from this analysis showed that melanoma patients with brain metastases who received ICI + SRS had significantly better overall survival comparing to those who had SRS only (HR = 0.66, 95% CI 0.53–0.83). Melanoma patients who had nonconcurrent therapy had significantly lower overall survival comparing to those who received concurrent ICI-SRS therapy (HR = 1.64, 95% CI 1.23–2.18).

In a meta-analysis, Tan et al. assessed clinical outcomes in patients from 41 observational studies and 12 clinical trials [58]. Overall survival was significantly better in patients who received immunotherapy + SRS versus those who received SRS alone (HR = 0.48, 95% CI 0.32–0.73) while this clinical benefit was not observed in patients who received SRS + targeted therapy with BRAF/MEK inhibitors versus those who received SRS alone (HR = 0.71, 95% CI, 0.35–1.44). Interestingly, results from the plot analysis failed to show survival benefit in patients who received SRS + immunotherapy + targeted therapy comparing to those who received SRS alone.

A pooled analysis of data from 35 studies including 13,959 patients receiving ICI alone and from 16 studies with 1,442 patients receiving ICI + radiotherapy showed no significant increased risk of grade 3-toxicities in patients who received ICI + radiotherapy comparing to those who received ICI alone (16.3% versus 22.3%) suggesting that that administration of ICI within 90 days after radiotherapy is safe [59].

An analysis of the data from a cohort of 450 patients from registry ADOREG demonstrated that SRS combined with systemic therapy is beneficial in patients with melanoma brain metastases [60]. Results from this multicenter study suggest that immunotherapy should be considered the first-line therapy while targeted therapy with BRAF/MEK inhibitors as second-line therapy.

Substantial evidence from conducted studies showed the potential synergy of SRS and immunotherapy or targeted therapy suggesting that their concurrent administration improves local tumour control and provides prolonged survival. However, currently there is no consensus on the standard use of this combined treatment modality. Although many melanoma centers select concomitant use of SRS and immunotherapy or targeted therapy in the management of advanced melanoma with brain metastases, the final decision on the choice of therapy should be individualised for each patient after careful multidisciplinary treatment planning in which the risk and all benefits should be evaluated jointly with the patient. More results from randomised control trials are required before combination of SRS and immunotherapy or targeted therapy becomes the standard of care for melanoma patients with brain metastases.

### Recommendations Regarding Use of WBRT Alone

Due to the lack of survival benefit and high neurocognitive toxicity, the role of WBRT has been questioned during the last decade.

According to the EORTC guideline WBRT should be abandoned from the treatment of melanoma brain metastases. EANO-ESMO guideline does not recommend the routine WBRT nor the use of WBRT after surgical resection of brain metastases or after SRS. This guideline discourages the routine use of WBRT in patients with multiple brain metastases not amenable to SRS, restricting it only to patients carefully selected based on neurological symptoms, size, number and location of brain metastases and the choice and availability of CNS-active systemic therapy. EANO-ESMO guideline does not support use of WBRT as palliative treatment. NCCN guideline does not recommend routine WBRT or WBRT after surgical resection or SRS of brain metastases. This US guideline considers WBRT for palliative purposes in patients with good KPS (Karnofsky Performance Scale) status who have failed systemic therapy and who are not suitable for SRS. Hippocampal avoidance and memantine therapy should be offered to patients who will receive palliative WBRT. This is in line with ASCO-SNO-ASTRO guideline that endorses WBRT only for patients with poor performance status or with metastases that are not suitable for SRS.

CCA guidelines suggests that WBRT may provide some palliative benefits for patients with multiple brain metastases.

## Conclusion

With the use of SRS as preferred first-line treatment for brain metastases and omission of WBRT for all patients, a significant shift in the clinical management of advanced melanoma has been observed. However, the optimal treatment for melanoma brain metastases still has to be determined. While awaited results from several prospective clinical trials evaluating concomitant use of SRS and immunotherapy will provide efficacy data of this combined treatment modality, further clinical research is required to generate answers regarding optimal timing for initiation of systemic therapy (before or after SRS) as well as optimal radiation dose to treat melanoma brain lesions. Findings from these investigations will help to define new standards and recommendations for the treatment of melanoma patients with brain metastases.

## Key References

- Williams, G.J., et al. Treatment of melanoma brain metastases with radiation and immunotherapy or targeted therapy: A systematic review with meta-analysis. Crit Rev Oncol Hematol 2024;202:104462.
  - ○ This reference is of outstanding importance because it provides a comprehensive review with meta-analysis on the use radiation and immunotherapy or targeted therapy for the treatment of melanoma brain metastases.
- Tan, X.L., et al. Current Treatment Approaches and Global Consensus Guidelines for Brain Metastases in Melanoma. Front Oncol 2022;12:885472.
  - ○ This reference is of outstanding importance because it systematically summarizes current treatment options and compare different guidelines for the treatment of melanoma brain metastases.
- Mandalà, M., et al. Combined immunotherapy in melanoma patients with brain metastases: A multicenter international study. Eur J Cancer 2024;199:113542.
  - ○ This reference is of importance because it presents results from a multicenter study showing significant survival benefits of patients treated with ipilimumab plus nivolumab (COMBO) outside clinical trials.

## Data Availability

No datasets were generated or analysed during the current study.
